# Discontinuation of Nucleos(t)ide Analog treatment in HBeAg-Negative Non-Cirrhotic Chronic Hepatitis B Patients: Real-Life Data of 20 Years

**DOI:** 10.5152/tjg.2023.22823

**Published:** 2023-11-01

**Authors:** Bilgül Mete, Sibel Yıldız Kaya, Abdurrahman Kaya, Ahmet Furkan Kurt, Osman Faruk Bayramlar, Rıdvan Karaali, İlker İnanç Balkan, Mücahit Yemişen, Reşat Özaras, Neşe Saltoğlu, Fehmi Tabak

**Affiliations:** 1 Department of Infectious Disease, İstanbul University–Cerrahpaşa Faculty of Medicine, İstanbul, Turkey; 2 Department of Infectious Disease, İstanbul Training and Research Hospital, İstanbul, Turkey; 3 Department of Public Health, Bakırköy District Health Directorate, İstanbul, Turkey; 4 Department of Internal Medicine, Medistate Hospital İstanbul, İstanbul, Turkey; 5 Department of Infectious Diseases, Medilife Health Group, İstanbul, Turkey

**Keywords:** Hepatitis B, treatment, discontinuation, relapse, re-treatment

## Abstract

**Background/Aims:**

Discontinuation of nucleos(t)ide analog is controversial in HBeAg-negative chronic hepatitis B patients not achieved HBsAg loss. We aimed to evaluate re-treatment rates and risk factors in non-cirrhotic HbeAg-negative chronic hepatitis B patients for whom nucleosi(t)ides analogs were discontinued.

**Materials and Methods:**

Demographic, clinical, and laboratory data before and at the end after discontinuation of nucleos(t)ide analogs were collected retrospectively.

**Results:**

Seventy-two patients followed up between January 2000 and December 2019 were included; 43 were male, with a mean age of 46.3 (±10.8). Baseline median alanine aminotransferase (ALT) and hepatitis B virus DNA levels were 55.5 IU/L and 465 925 IU/mL, respectively. The median histologic activity index was 5.5 and the fibrosis score was 2. The median duration of treatment and consolidation therapy were 59 and 56 months, respectively. The median follow-up time after discontinuation of treatment was 55 months. Among 56 patients eligible for evaluation according to proposed re-treatment criteria, 29 (51.7%) patients were re-treated. The median time for relapse was 11 months. Re-treatment was significantly common in males (*P* = .034) and patients treated with tenofovir/entecavir (*P* = .04). Baseline hepatitis B virus DNA and levels of ALT, aspartate aminotransferase (AST) at the third and sixth months of treatment and at the end of treatment were statistically significantly higher in re-treated patients. A cutoff value of ≥405 000 IU/L for hepatitis B virus DNA discriminated patients for re-treatment. HBsAg was lost permanently in 2 non-re-treated patients.

**Conclusion:**

In resource-limited areas where follow-up of HBsAg or other markers is not possible, nucleos(t)ide analog discontinuation can be considered in patients in the early stage, with low baseline hepatitis B virus DNA and ALT levels, after a long consolidation therapy.

**Main Points**

- Nucleos(t)ide analog discontinuation can be considered in patients: (1) with Ishak’s fibrosis score 1-3, (2) low baseline hepatitis B virus DNA at admission, and (3) after a long consolidation therapy (3-5 years) and close follow-up.
- Re-treatment is required in approximately half of the patients.
- HBsAg loss is higher in non-re-treated patients.

## Introduction

Long-term treatment with high genetic barrier nucleosi(t)ides analogs (NUC) such as entecavir or tenofovir leads to suppression of hepatitis B virus (HBV) replication, improvement in liver histopathology, reduction in risk of cirrhosis and hepatocellular carcinoma (HCC), and improvement in overall survival.^1^

Due to the risk of relapse after discontinuation of antiviral therapy, long-term even life-long treatment has been considered.^1^ The recommended endpoint for treatment is HBsAg loss with or without subsequent seroconversion in all subgroups of patients.^2-4^ However it has been demonstrated in some studies that virological and biochemical remissions are sustained, liver histology does not progress, and the risk of HCC does not increase after cessation of antiviral therapy.^1,5,6^ Although the side effects of NUCs are very few, safety data of HBV drugs are limited to 8-10 years, cumulative cost and decrease in drug adherence over time are among the other disadvantages of life-long treatment.^1^ Based upon these reasons, discontinuation of HBV therapy was first proposed in Asian-Pacific Association for Study of the Liver (APASL), and treatment-stopping criteria were determined for patients with HbeAg-negative chronic hepatitis B (CHB) in 2008 APASL with concerns of cost and resistance.^7^

According to current recommendations of the major guidelines, treatment can be discontinued in HBeAg-positive non-cirrhotic CHB patients after HBeAg seroconversion and HBV DNA undetectability, with at least 1-3 years of consolidation therapy. For HBeAg-negative non-cirrhotic CHB patients, treatment discontinuation may be considered after at least 2-3 years of therapy with undetectable HBV DNA levels (on 3 separate occasions 6 months apart). Consolidation therapy was defined as a treatment after the first undetectable HBV DNA (and HBeAg loss for HBeAg-positive patients) until NUC discontinuation.^2-4^

In this study, we aimed to evaluate HBeAg-negative non-cirrhotic CHB patients in whom NUCs were discontinued for any reason and to analyze virological and/or clinical relapse rates, risk factors for re-treatment, and re-evaluate the re-treated patients according to newly proposed re-treatment strategies.

## Materials and Methods

We retrospectively included ≥18-year-old chronic HBeAg-negative patients followed-up by our outpatient clinic between January 2000 and December 2019 and for whom HBV treatment was discontinued for any reason. Exclusion criteria were co-infection with hepatitis C virus, and/or hepatitis delta virus and/or human immunodeficiency virus, immunosuppressive therapy, cirrhosis, treatment with interferons, treatment and/or follow-up after discontinuation of treatment for less than 1 year. The study was approved by the Committee of Ethics of İstanbul University-Cerrahpaşa (E-83045809-604.01.01-526443). Informed consent could not be obtained since it was a retrospective study.

Demographic, biochemical, serological, and virological laboratory data and fibrosis stage of the liver at the time of diagnosis, NUC administration, duration of treatment and consolidation therapy, biochemical, serological, and virological laboratory data, during and at the end of treatment and after discontinuation of NUCs, quantitative HBsAg levels (if measured) at the end of treatment were collected from the files and computer records, retrospectively.

Fibrosis and necroinflammation scores were assessed histologically using Ishak’s scoring system on liver biopsies at the time of diagnosis.

The patients were evaluated according to the definitions cited below:

Virological relapse was defined as HBV DNA >2000 IU/mL; biochemical relapse was defined as ALT >2 times upper limit of normal (ULN) in addition to virological relapse.^4^

Re-treated patients were re-analyzed if treatment was essential according to newly proposed re-treatment criteria:^1,8^

### Severe Flares

ALT levels >10 × ULN, or ALT >5 × ULN combined with increased bilirubin levels (>2 mg/dL) and/or prolongation of prothrombin time.

### Persistent Mild-to-Moderate Disease Activity

Persistently elevated ALT (>2 times ULN) and HBV DNA >2000 IU/mL for at least 3-6 months or modest ALT elevations (>3 × ULN) and HBV DNA levels >100 000 IU/mL concomitantly.

Patients requiring re-treatment were defined as clinical relapser in this study. 

Sustained response was defined as persistent HBV DNA <2000 IU/mL after treatment cessation. HBsAg loss was confirmed on 2 consecutive samples at least 1 month apart.

HBsAg levels were quantified by Elecsys HBsAg II Quant kits (Roche Diagnostics, Indianapolis, IN, USA) according to the manufacturer’s instructions.

### Statistical Analysis

Continuous variables were presented with mean ± SD or median and interquartile range (IQR) based on their distribution. Categorical variables were shown with frequencies and percentages. Continuous variables were evaluated for normality distribution using Shapiro–Wilk test. Categorical variables were compared by using chi-square or Fisher’s exact test for proportion. For 2-group comparison, independent sample *t*-test was used for normally distributed variables and Mann–Whitney *U* test for nonnormally distributed variables. Univariate logistic regression analysis was performed to evaluate the association between the occurrence of relapse risk and independent factors, and multivariate logistic regression was performed with variables found to be statistically significant. Odds ratio (OR) and their 95% CI were presented. Baseline HBV DNA was highly skewed, therefore we applied natural log transformation on baseline HBV DNA. Parameter estimates were more satisfied after the natural log-transformed on baseline HBV DNA. In order to assess the diagnostic performance of baseline HBV DNA, baseline HBs Ag, and baseline ALT in determining the occurrence of relapse, receiver operating characteristic curve (ROC) analysis was performed. The optimal cut point was obtained by maximizing the sensitivity and specificity. All significant tests were 2-tailed, and values of *P* < .05 were considered statistically significant. All statistical analyses were performed by Statistical Package for the Social Sciences software version 21.0 (IBM Corp.; Armonk, NY, USA).

## Results

Of the 72 patients included in the study, 43 (59.7%) were male, with a mean age of 46.3 (±10.8) years. Baseline median ALT and HBV DNA levels were determined as 55.5 IU/L (IQR: 26.25-107.5) and 465 925 IU/mL (IQR: 46 700-2 868 810), respectively. Liver biopsy was performed in 97% of the patients; the median necroinflammation score was 5.5 (IQR: 4-8) and the fibrosis score was 2 (IQR: 1-2). The median duration of NUC treatment and consolidation therapy was 59 months (IQR: 3-6) and 56 months (IQR: 18-69), respectively. Lamivudine, entecavir, and tenofovir were administered in 33.2%, 16.1%, and 46.4% of the patients, respectively. Treatment was discontinued in 50 (69.4%) patients upon the decision of the physician, and 17 patients (23.6%) self-discontinued the NUCs. In the remaining cases, the reasons for discontinuation of NUCs were pregnancy in 2 and side effects in 3 patients. Nucleos(t)ide analog was discontinued in 5 patients before HBV DNA was undetectable. The median follow-up time at the end of treatment was 55 months (IQR: 37-81). The mean follow-up after discontinuation of treatment was 71.3 ± 40.1 months.

The baseline characteristics of the patients were demonstrated in Table 1.

**Figure 1.**
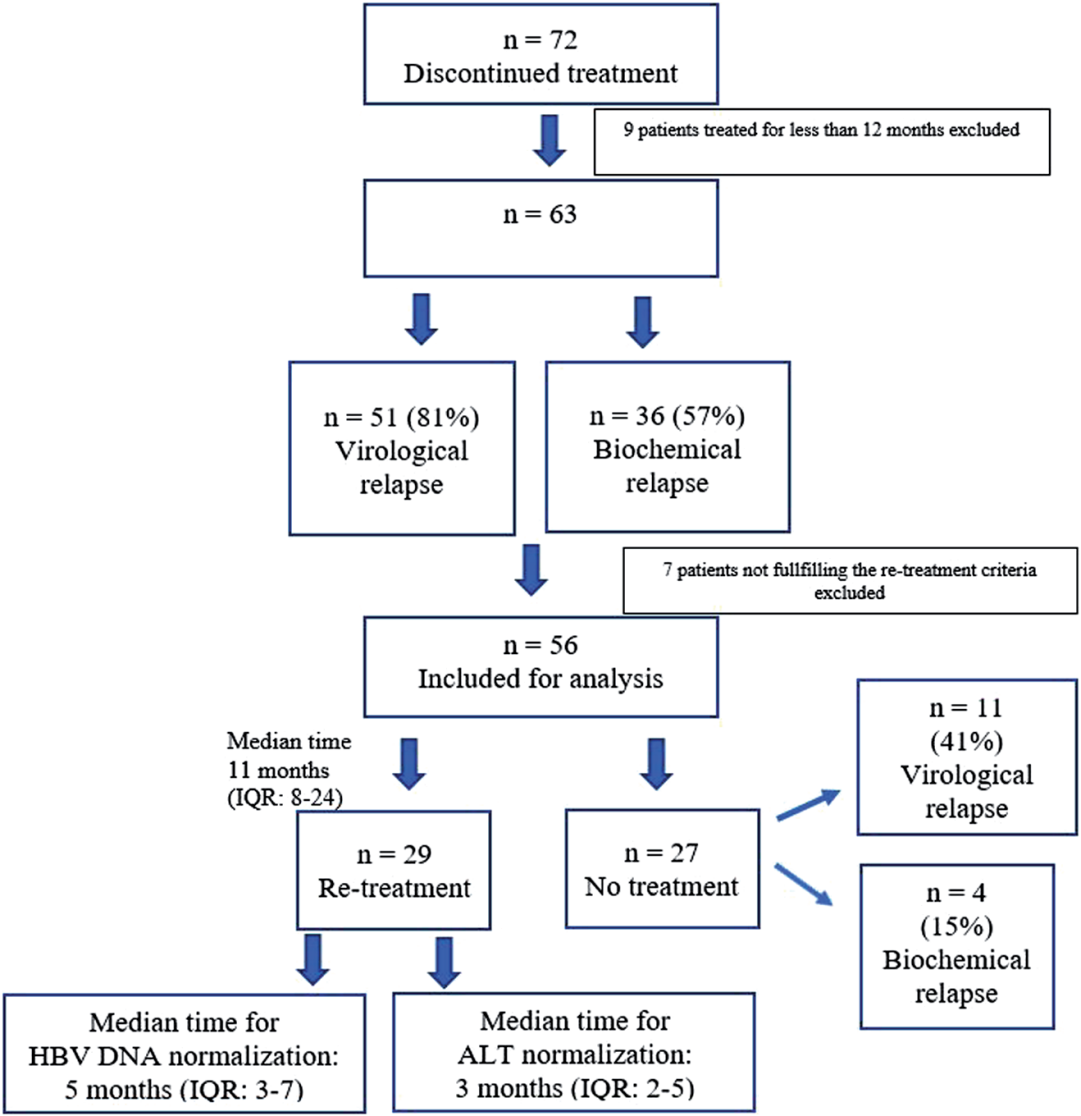
Analysis of patients discontinuing nucleos(t)ide analogs.

Nine patients self-discontinued the NUCs before 12 months (median time: 6 months) and thus not evaluated in the analysis. Sixty-three patients were included for further analysis. Virological relapse developed in 80.9% and biochemical relapse developed in 57.1% of them. Detailed analysis revealed that 7 patients were re-treated but when re-evaluated according to the proposed re-treatment criteria, it was determined that they do not fulfill these criteria. Therefore these patients were also excluded, and finally, 56 patients were included for analysis of re-treatment due to relapse (Figure 1). According to the relevant criteria, re-treatment was required in 29 (51.7%) of the patients and 44.8% of them developed severe flare but all recovered. The median time for HBV DNA and ALT normalization after re-treatment was 5 months (IQR: 3-7) and 3 months (IQR: 2-5), respectively. Only 1 relapser was lost due to HCC: he self-discontinued the treatment and was not available for follow-up due to COVID-19 pandemic. About 40.7% and 14.8% of the patients who were not re-treated developed virological and biochemical relapse, respectively.

The median time for relapse was 11 months (IQR: 8-24) in re-treated patients and 1 patient relapsed after 6 years. It was observed that 82.7% of the cases relapsed within the first 6 months after discontinuation of treatment. When compared with those who were not re-treated, there was no significant difference in terms of age, necroinflammatory/fibrosis scores, and duration of treatment. Re-treatment was significantly more common in males (*P* = .034) and in patients treated with tenofovir/entecavir compared with telbivudine and lamivudine (*P* = .04). Baseline HBV DNA and levels of ALT, AST at third and sixth months of treatment and at the end of the treatment were found statistically significantly higher in re-treated patients than those who did not (Tables 2 and 3).

The univariate analysis revealed that except for levels of ALT, AST at third month, all parameters mentioned above were risk factors for re-treatment. A multivariate logistic regression model was studied with the variables that were significant in the univariate logistic regression analysis, and gender, baseline HBV DNA levels, treatment method, ALT and AST levels at the sixth month of treatment, and ALT and AST levels at the end of treatment were the risk factors that affect clinical relapse in the patients (Table 4).

The relapsers were subgrouped according to the relapse time for further analysis. Although the results were insignificant, consolidation therapy duration was longer in the group relapsing after 6 months compared to the group relapsing within 6 months.

When 18 patients whose HBsAg levels were measured quantitatively before drug discontinuation were analyzed, no significant difference was present in clinical relapsers versus nonrelapsers (*P* = .930).

Receiver operator characteristic curve analysis was performed to determine cutoff values for HBV DNA and HBsAg levels. Baseline HBV DNA level was found to be an important variable in identifying patients with clinical relapses and a cutoff value of ≥405 000 IU/L for HBV DNA was demonstrated to discriminate clinical relapse. The HBsAg level was not found to be a statistically significant variable in discriminating patients with relapse. But when the sensitivity and specificity ratios were examined, the most appropriate cutoff point was determined as ≥1700 IU/mL.

HBsAg was lost in 4 non-clinical relapsers (14.8%) and 2 of them developed HBs seroconversion. HBsAg converted reversely in 2 patients who did not seroconvert (5 years after in 1 patient and 8 years after in the other).

## Discussion

Long-term even life-long treatment is still the recommended modality for patients with CHB. However, concerns about adherence to the treatment and economic burden especially in resource-limited regions and data from studies revealing that virological and clinical remission are sustained in non-cirrhotic patients after cessation of treatment led to the development of NUC stopping rules in selected patients.^1-7^ However discontinuation of NUCs is still a controversial issue.

Hadziyannis et al^9^ first demonstrated that sustained virological and biochemical remission was observed in 55% of the HBeAg-negative CHB patients after discontinuation of treatment and 72% of these patients had HBsAg loss. Although relapse/remission definitions and follow-up periods vary according to studies, virological and clinical relapse rates in HBeAg-negative CHB range between 30%-70% and 35%-65%, respectively.^10,11^ The biochemical relapse rate in our series was 57.1% with a median follow-up duration of 55 months. But re-treatment rate was relatively low (51.7%). The median duration of NUC treatment and consolidation therapy was 59 and 56 months, respectively. Considering that total antiviral and consolidation treatment durations were long, we think that these results may be considered reliable and NUCs may be discontinued in selected patients.

One of the important concerns in treatment cessation is the risk of development of HCC. However, it is demonstrated that the risk of HCC decreases after 5 years of treatment.^12^ The median total NUC treatment duration was approximately 5 years in our study.

Most of the relapses occur within 3-9 months after discontinuation and decrease after 6 months.^8,11^ In our study, the median time for relapse was 11 months and we observed that 82.7% of the relapses occurred in the first 6 months.

The most common predictors of relapse are older age, male sex, genotype, late stage of the disease, type of NUC, short consolidation therapy duration, baseline and end of treatment levels of ALT, HBV DNA, and HBsAg.^8,10,13^

Male gender was also a significant predictor of relapse in our study, and it was observed that 69% of the relapses developed in male patients.

The probability of functional cure is higher in Caucasians after discontinuation of NUCs and this may be associated with the genotype.^14^ Since genotype D is predominant in our country, relatively high rate of functional cure may be partly due to this factor.^15^

The fibrosis scores (median = 2) were similar and low in our patients, therefore the effect on relapse could not be evaluated.

Tenofovir was demonstrated to be associated with a higher and earlier risk of relapse compared with entecavir.^9^ In our study, 62.1% of the re-treated patients were treated with tenofovir and the relapse rate was higher in patients treated with tenofovir/entecavir compared to lamivudine/telbivudine. Tenofovir and entecavir provide sustained viral suppression owing to their better resistance barriers than lamivudine and telbivudine. This unexpected result may be explained by the tendency of physicians to prefer tenofovir or entecavir for severe cases.

Short consolidation therapy duration is correlated with relapse and the guidelines recommend at least 2-3 years of consolidation treatment.^2-4,16^ Since the median consolidation therapy duration in our study was relatively long, no significant difference was detected within subgroups.

The role of ALT is controversial but lower baseline ALT levels may be associated with a higher rate of HBsAg loss.^17,18^ Baseline liver enzyme levels (*P* = .057) and levels at third, sixth month, and at the end of the treatment were significantly lower in non-relapsers in our study. However, multivariate logistic regression analysis revealed that only ALT levels at sixth month of treatment significantly affected the rate of clinical relapse.

In most of the studies, lower baseline HBV DNA levels predicted virological remission.^4,10,13,17^ Jeng et al^19^ demonstrated in their study that baseline HBV DNA level <2 × 105 IU/mL predicted sustained response after cessation of NUC treatment. Baseline HBV DNA levels were significantly lower in non-re-treated patients in our series. Receiver operating characteristic curve analysis demonstrated that a cutoff value of ≥405 000 IU/mL may predict the risk of clinical relapse in our study.

Higher HBsAg levels at baseline and end of treatment may predict the probability of relapse.^1,4,13,16^ Liu et al^20^ in their systemic review determined that besides old age and shorter duration of consolidation therapy, end of treatment HBsAg levels ≥1000 IU/mL were predictive factors of relapse in HBeAg-negative CHB patients. They also demonstrated that in case of HBsAg level <100 IU/mL, the virological and clinical relapse rate was 9%-19% and 15%-29%, respectively. In the study of Ge et al,^21^ HBsAg level ≥1500 IU/mL at the end of the treatment was the only predictive factor for relapse. In our country, quantitative HBsAg level measurement is not reimbursed. Only 18 patients had quantitative HBsAg levels at the end of treatment. HBsAg level of less than 100 IU/mL was not detected in any patient in our study. In limited number of patients with HBsAg level, our study did not find it as a significant determinant discriminating the relapse.

The HBsAg loss is greatest in patients with sustained response who were not re-treated in case of relapse and lowest in those re-treated.^22^ HBsAg loss rate is higher in Caucasian patients compared to the Asian population.^23^ Although genotype D is prevalent in our country, the sustained rate of HBsAg loss was relatively low compared to other reports (7.4%) in patients who were not re-treated.

In conclusion, in resource-limited areas where follow-up of HBsAg or other markers is not possible, NUC discontinuation can be considered in patients admitted in the early stage (Ishak’s fibrosis score: 1-3) with low baseline HBV DNA (<400 000 IU/mL) and ALT levels, after a long consolidation therapy (3-5 years) and close follow-up. Re-treatment is required in approximately half of the patients and biochemical remission is achieved within 3 months. HBsAg loss is higher in non-re-treated patients.

## Figures and Tables

**Table 1.** Baseline Characteristics of the Patients

Patients	n = 72
Age (years, mean ± SD)	46.33 ± 10.8
Sex (n, %)	
Male	43 (59.7)
Female	29 (40.3)
Baseline median ALT (U/L) (IQR)	55.5 (26.25-107.5)
Baseline median AST (U/L) (IQR)	39 (23.5-60)
Baseline mean albumin (mg/dL) (±SD)	4.2 ± 0.4
Baseline median total bilirubin (mg/dL) (IQR)	0.64 (0.48-0.89)
Baseline median HBV DNA (IU/mL) (IQR)	465 925 (46 700-2 868 810)
Ishak fibrosis score, median (IQR)	2 (1-2)
Ishak necroinflammation score, median (IQR)	5.5 (4-8)
NUC, n (%)	
Tenofovir	26 (46.4)
Entecavir	9 (16.1)
Lamivudine	18 (33.2)
Telbivudine	2 (3.6)
Adefovir	1 (1.8)
Time to undetectable HBV DNA (months), median (IQR)	6 (3-6)
Consolidation therapy duration (months), median (IQR)	56 (18-69)
Total NUC treatment time (months), median (IQR)	59 (18.5-73.75)
EOT follow-up time (months), median (IQR)	55 (37-81)

EOT, end of treatment; HBV, hepatitis B virus; IQR, interquartile range; NUC, nucleos(t)ide analogs.

**Table 2.** Effect of Baseline Virological and Biochemical Parameters and Treatment Modalities on Relapse

	Re-Treatment	No re-treatment	Total	*P*
	n = 29	n = 27	n = 56	
Age (years)	47.0 ± 9.5	47.6 ± 10.2	47.3 ± 9.7	.833
Sex, n (%)				
Male	20 (69.0)	11 (40.7)	31 (55.4)	**.034**
Female	9 (31.0)	16 (59.3)	25 (44.6)	
Baseline median ALT (U/L) (IQR)	74 (42.0-127.5)	30 (20.0-96.0)	51 (25.25-113.75)	.057
Baseline median AST (U/L) (IQR)	42 (26.5-69)	28 (20-54)	36 (21.5-59)	.076
Baseline mean albumin (mg/dL)	4.1 ± 0.4	4.3 ± 0.3	4.2 ± 0.5	.148
Baseline median total bilirubin (mg/dL) (IQR)	0.6 (0.49-0.89)	0.78 (0.5-0.9)	0.67 (0.5-0.89)	.749
Baseline median HBV DNA (IU/mL) (IQR)	1 908 869 (408 425-204 000 000)	55 000 (15 460- 580 155)	535 577.5 (46 700-2 868 810)	**<.001**
Ishak fibrosis score, median (IQR)^*^	2 (1-2.5)	2 (1-2)	2 (1-2)	.147
Ishak necroinflammation score, median (IQR)^**^	6.0 (9.0-4.0)	5.0 (7.0-4.0)	5.5 (4-7.75)	.480
NUC, n (%)				
Tenofovir	18 (62.1)	8 (29.6)	26 (46.4)	.040*
Entecavir	6 (20.7)	3 (11.1)	9 (16.1)	
Lamivudine	5 (17.2)	13 (48.2)	18 (32.1)	
Telbivudine	0 (0.0)	2 (7.4)	2 (3.6)	
Adefovir	0 (0.0)	1 (3.7)	1 (1.8)	
Time to undetectable HBV DNA (months), median (IQR)	6.0 (3.0-8.75)	4.0 (3.0-6.0)	6 (3-6.5)	.184
Consolidation therapy duration (months), median (IQR)	61.5 (31.5-77.0)	57 (27.5-67.5)	59 (3370)	.382
Total NUC treatment time, (months), median (IQR)	68 (34-80)	63.0 (25-73)	65 (24-75)	.231

^*^Tenofovir vs. telbuvidine and tenofovir vs. lamivudine.

HBV, hepatitis B virus; IQR, interquartile range; NUC, nucleos(t)ide analogs. Bold values are statistically significant (*P* < .05).

**Table 3.** Effect of Virological and Biochemical Parameters During and at the End of Treatment on Relapse

	Re-treatment	No re-treatment	Total	*P*
	n = 29	n = 27	n = 56	
Median ALT (U/L), third month of treatment (IQR)	34 (23.5-48.5)	22 (17-19)	26.5 (19.25-42.25)	**.007**
Median AST (U/L), third month of treatment (IQR)	25 (21.5-32)	20 (17-25)	22 (17-29.5)	**.024**
Median total bilirubin (mg/dL), third month of treatment (IQR)	0.73 (0.53-0.98)	0.54 (0.44-0.80)	0.62 (0.45-0.93)	.123
Median HBV DNA (IU/L), third month of treatment (IQR)	430 (0-2188.5)	315 (0-2250)	372.5 (0-2249.75)	.933
Median ALT (U/L), sixth month of treatment (IQR)	29 (21-40.5)	18 (16-22)	22 (18-31)	**<.001**
Median AST (U/L), sixth month of treatment (IQR)	25 (21-29)	19 (18-22)	22 (18-27.75)	**.001**
Median total bilirubin (mg/dL), sixth month of treatment (IQR)	0.63 (0.52-0.93)	0.45 (0.40-0.75)	0.59 (0.41-0.82)	.108
Median HBV DNA (IU/L), sixth month of treatment (IQR)	0 (0-134.5)	0 (0-0)	0 (0-114.75)	.371
EOT ALT (U/L), median (IQR)	25 (18-33)	18 (14-24)	20 (16-31.8)	**.006**
EOT AST (U/L), median (IQR)	23 (18-26.5)	18 (14-21)	20 (16-24.75)	**.002**
EOT total bilirubin (mg/dL), median (IQR)	0.46 (0.29-0.8)	0.48 (0.4-0.6)	0.47 (0.36-0.68)	.762
EOT HBV DNA (IU/L), median (IQR)	0 (0-0)	0 (0-0)	0 (0-0)	.488
EOT HBsAg (IU/mL), median (IQR)^*^	2255 (1236-4860)	1958 (1369-3323)	2106.5 (1298.25-3439)	.930

^*^Eighteen patients were evaluated: 11 in clinical relapse, 7 in no relapse group.

EOT, end of treatment; HBV, hepatitis B virus; IQR, interquartile range; NUC, nucleosi(t)ides analogs. Bold values are statistically significant (*P* < .05).

**Table 4.** Logistic Regression Analysis of Risks Factors for Clinical Relapse

	Univariate Analysis		Multivariate Analysis	
	OR (95% CI)	*P*	OR (95% CI)	*P*
Sex	0.309 (0.103-0.929)	**.036**	0.106 (0.012- 0.934)	**.043**
Baseline HBV DNA (IU/mL) (log_e_ IU/mL)	1.627 (1.241-2.132)	**<.001**	2.038 (1.264-3.284)	**.003**
NUC^*^	0.143 (0.042-0.491)	**.002**	0.059 (0.004-0.781)	**.032**
ALT level (IU/L) at third month of treatment	1.00 (0.987-1.02)	.709	—	—
AST level (IU/L) at third month of treatment	0.997 (0.972-1.022)	.819	—	—
ALT level (IU/L) at sixth month of treatment	1.112 (1.035-1.195)	**.004**	1.256 (1.051-1.501)	**.012**
AST level (IU/L) at sixth month of treatment	1.143 (1.032-1.265)	**.010**	0.757 (0.582-0.984)	**.037**
EOT ALT level (IU/L)	1.076 (1.01-1.147)	**.024**	0.912 (0.762- 1.091)	.314
EOT AST level (IU/L)	1.195 (1.053-1.356)	**.006**	1.536 (1.057- 2.233)	**.024**

^*^Tenofovir vs. telbuvidine and tenofovir vs. lamivudin.

EOT, end of treatment; HBV, hepatitis B virus; IQR, interquartile range; NUC, nucleos(t)ide analogs; OR, odds ratio. Bold values are statistically significant (*P *< .05).
